# Soybean Trypsin Inhibitor Possesses Potency Against SARS-CoV-2 Infection by Blocking the Host Cell Surface Receptors ACE2, TMPRSS2, and CD147

**DOI:** 10.3390/ijms26146583

**Published:** 2025-07-09

**Authors:** Wen-Liang Wu, Jaung-Geng Lin, Wen-Ping Jiang, Hsi-Pin Hung, Atsushi Inose, Guan-Jhong Huang

**Affiliations:** 1 School of Chinese Medicine, College of Chinese Medicine, China Medical University, Taichung 404, Taiwan; boss-16888@hotmail.com (W.-L.W.); jglin@mail.cmu.edu.tw (J.-G.L.); pin721.hwatou@gmail.com (H.-P.H.); 2 Chinese Medicine Research Center, China Medical University, Taichung 404, Taiwan; 3 Department of Pharmacy, China Medical University, Taichung 404, Taiwan; wpjiang@cmu.edu.tw; 4 Faculty of Pharmacy, Nihon Pharmaceutical University, 10281, Komuro, Ina-machi, Kita-Adachi-gun, Saitama 362-0806, Japan; ainose@nichiyaku.ac.jp; 5 Department of Chinese Pharmaceutical Sciences and Chinese Medicine Resources, College of Chinese Medicine, China Medical University, Taichung 404, Taiwan; 6 Department of Food Nutrition and Healthy Biotechnology, Asia University, Taichung 413, Taiwan

**Keywords:** soybean trypsin inhibitor, SARS-CoV-2, ACE2, TMPRSS2, CD147

## Abstract

Angiotensin-converting enzyme 2 (ACE2) is a cell-surface receptor that helps the body regulate blood pressure and endocrine secretions. Transmembrane serine protease 2 (TMPRSS2) is a cell surface protein expressed mainly by endothelial cells of the respiratory and digestive tract, which participates in the cleavage of protein peptide bonds with serine as the active site. These two proteins have been studied to be highly associated with infection with severe acute respiratory syndrome coronavirus 2 (SARS-CoV-2). Soybean trypsin inhibitor (SBTI) has special bioactivities such as anticarcinogenic and anti-inflammatory functions, which can be widely used in functional foods or drugs. Our study involved in vitro and in vivo experiments to elucidate the effect of SBTI on SARS-CoV-2 host invasion. First, it was confirmed that being under 250 μg/mL of SBTI was not toxic to HepG2, HEK293T, and Calu-3 cells. The animal study administered SBTI to mice once daily for 14 days. In the lungs, liver, and kidneys, the histopathologic findings of the SBTI group were not different from those of the control group, but the expression of ACE2, TMPRSS2, and CD147 was reduced. Thus, our findings suggest that the inhibition of ACE2, TMPRSS,2 and CD147 proteins by SBTI shows promise in potentially inhibiting SARS-CoV-2 infection.

## 1. Introduction

The coronavirus disease 2019 (COVID-19) caused by the SARS-CoV-2 virus has led to a major global epidemic affecting many levels [1]. Today, the evolution of the virus is still ongoing, and there is a great deal of concern as to whether it will gradually “flu-ize” or trigger another, more threatening pandemic. Effective vaccines and antiviral drugs will be essential for the response. Although vaccination as a preventive measure is feasible, much evidence remains that the infection rate remains high [2,3]. Antiviral drugs are currently an essential type of drug in international research and development. Although several therapeutic options have been proposed and tested, most require reliable clinical trial data before being used widely and safely. The genotype and gene expression status of ACE2 and TMPRSS2 in each individual may be key factors in determining susceptibility to SARS-CoV-2 infection [4,5]. Therefore, we would like to find a natural-source ingredient as a functional food or drug to prevent viral infection by studying the reduction of ACE2 and TMPRSS2 expression.

ACE2 is an enzyme that attaches to cell membranes in the intestines, kidneys, testes, gallbladder, and heart, and it is also found in soluble form [6]. Research has shown that the surface spike protein of the SARS-CoV-2 virus binds to ACE2 receptors, facilitating its entry into the human body [7]. TMPRSS2 is a cell surface protein expressed mainly by endothelial cells in the respiratory and digestive tracts, which participates in the cleavage of proteolytic peptide bonds with serine as the nucleophilic amino acid in the active site. TMPRSS2 activates spike proteins to bind to various proteins in host cells and assists the SARS-CoV-2 virus to invade the human body [8]. When the virus enters a human cell and strips off its membrane, the RNA of the virus, which carries the genetic message, will be released. Next, the RNA is translated into a polyprotein containing proteases that can cleave the polyprotein into various functionally distinct proteins, such as the RNA-dependent RNA polymerase, which is involved in the viral replication process [9]. Therefore, antiviral drugs aim to find targets to stop viral replication, including inhibiting the protease responsible for cleavage, inhibiting replication, or stopping gene replication through nucleotide compounds [10]. Trypsin, a prototypical serine endopeptidase, has been extensively researched for its ability to activate viral glycoproteins and has been demonstrated to activate the SARS-CoV-2 virus in vitro [11]. It has been suggested that inhibition of trypsin by inhalation of the FDA-approved drug aprotinin may help alleviate SARS-CoV-2 infections in the lungs [12]. CD147, a transmembrane adhesion molecule, plays a pivotal role in regulating inflammatory and immune processes. Recent studies, including our own, have identified CD147 as a universal receptor for the SARS-CoV-2 spike protein, facilitating viral entry across host cell types and variants. In addition to its role in viral infection, CD147 contributes to the pathogenesis of cytokine storm syndromes (CSSs) observed in severe COVID-19 by modulating cyclophilin A (CyPA) expression. These syndromes, characterized by excessive production of proinflammatory cytokines and chemokines, result in extensive tissue damage and multiorgan dysfunction. Mechanistically, the CD147-CyPA interaction activates ERK signaling pathways that amplify inflammatory responses [13]. Therapeutic targeting of this pathway has demonstrated anti-tumor efficacy, and the genetic or pharmacological suppression of CD147 impedes SARS-CoV-2 infection and replication. These multifaceted roles underscore CD147 as a promising and versatile target for the development of anti-inflammatory and antiviral therapies, particularly in the context of COVID-19 [14]. However, more evidence on the effects of trypsin inhibitors on other organs is still needed.

Among plants, trypsin inhibitor is a natural defense-associated protein that effectively protects against predators and pathogens by inhibiting proteases in the digestive tract [15]. Regarding antivirals, trypsin inhibitors have been studied for their activity in inhibiting human immunodeficiency virus proliferation [16]. The potential of trypsin inhibitors to target protease inhibition has been recognized as an effective treatment for SARS-CoV-2 [17,18]. Trypsin inhibitors are commonly found in Leguminosae. Soybean trypsin inhibitor (SBTI) belongs to the family of serpins, and there is evidence that SBTI features special biological activities, such as its anticancer and anti-inflammatory functions, which serve various applications [19]. In summary, we expect that SBTI, as a naturally derived trypsin inhibitor, has the potential to inhibit SARS-CoV-2 infection via ACE2, TMPRSS2, and CD147 pathways, which provides evidence for the development of a prospective functional food for COVID-19 therapy.

## 2. Results

### 2.1. Confirmation of the Effect on Cell Growth Using Different Concentrations of SBTI

**Figure 1:**
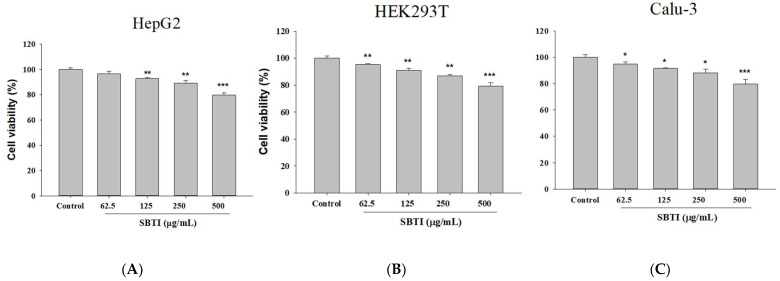
Cell viability after 24 h of co-culturing with different doses of SBTI. We used (**A**) HepG2, (**B**) HEK293T, and (**C**) Calu-3 cells to observe the results. At 250 μg/mL SBTI, cell viability exceeded 80% for both cells. Data are expressed as mean ± S.D. (*n* = 3). * *p* < 0.05, ** *p* < 0.01 and *** *p* < 0.001 compared to the control.

We first performed in vitro experimental screening of SBTI antiviral assays to minimize the use of experimental animals. HepG2, HEK293T, and Calu-3 cells can be screening models to study SARS-CoV-2 entry into host cells [20,21]. To ensure the safety of SBTI in the subsequent experiments, we first evaluated its cell toxicity using the MTT method (Figure 1). The results confirmed that SBTI concentrations below 250 μg/mL were non-toxic to both cells, so we chose 125 and 250 μg/mL for the next experiments.

### 2.2. Expression of ACE2, TMPRSS2, and CD147 Induced by SBTI in the In Vitro Experiment

**Figure 2:**
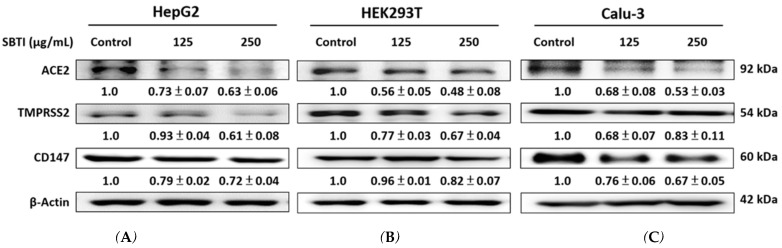
Measurement of ACE2, TMPRSS2, and CD147 levels in cells by Western blot analysis. Results showed that SBTI inhibited ACE2, TMPRSS2, and CD147 in (**A**) HepG2, (**B**) HEK293T, and (**C**) Calu-3 cells. Western blot analysis was used to quantify the protein expression of these proteins in the cells. Densitometric analysis was performed to quantify the protein bands. Each experiment was repeated independently at least three times, and representative images were shown. Each result is shown as mean ± S.D. Protein levels were quantified and displayed as fold changes relative to β-actin, which acted as the internal control for normalization.

The protein expression of ACE2, TMPRSS2, and CD147 in HepG2, HEK293T, and Calu-3 cells is shown in Figure 2, which initially demonstrated the antiviral entry effect of SBTI. SBTI treatment for 24 h led to a reduction in ACE2, TMPRSS2, and CD147 expression in HepG2, HEK293T, and Calu-3 cells, respectively—proteins associated with SARS-CoV-2 entry and modulation.

### 2.3. Evaluation of the Effect of SBTI on Animal Models

**Figure 3:**
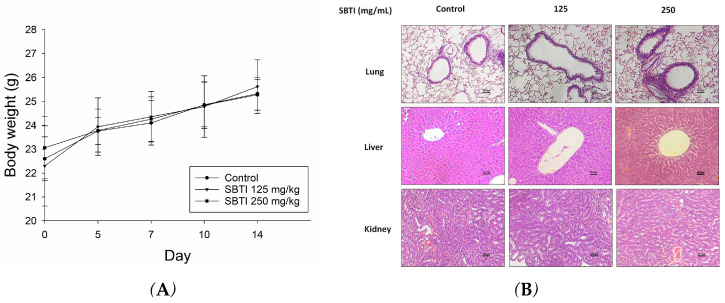
The inhibitory effect of SBTI was further analyzed with a mouse model. The results showed (**A**) the average body weight on days 0, 5, 7, and 14 and (**B**) the effects of H&E staining on lungs, livers, and kidneys observed under a 200× microscope. There were no significant differences in these results between the low-dose and high-dose SBTI groups compared to the control group. Data are expressed as mean ± S.D. (*n* = 6).

Next, we evaluated the effects of SBTI in vivo using a mouse model. Based on the findings, there was no significant difference in body weight compared to the untreated group when orally administering either 125 or 250 mg/kg of SBTI for 14 days (Figure 3A), and H&E staining showed that it did not cause any toxicity in the lungs, liver, or kidneys (Figure 3B).

### 2.4. Observing the Inhibitory Effects of SBTI on the Expression of ACE2, TMPRSS2, and CD147 Proteins by the IHC Method

**Figure 4:**
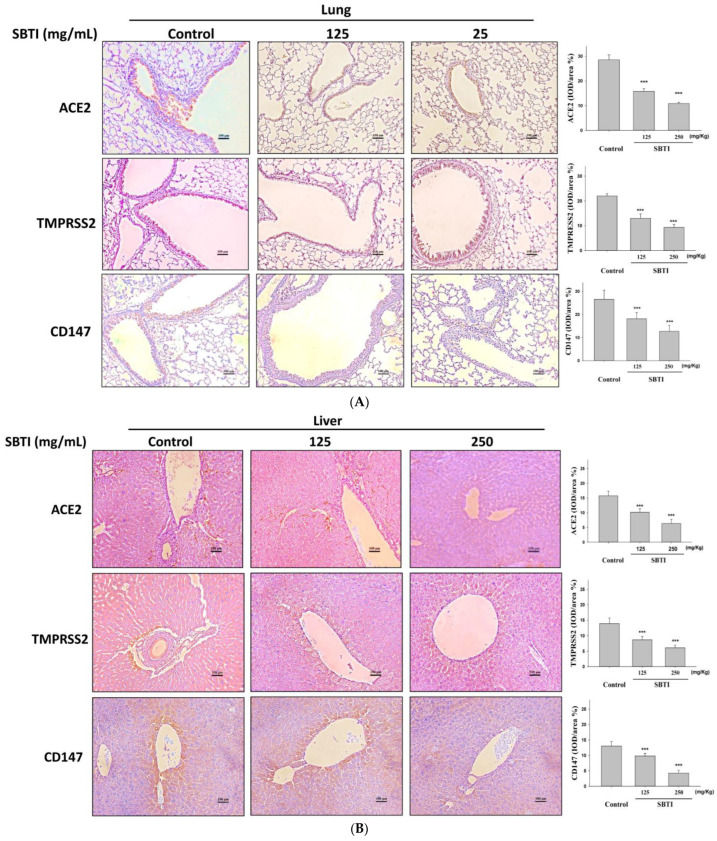

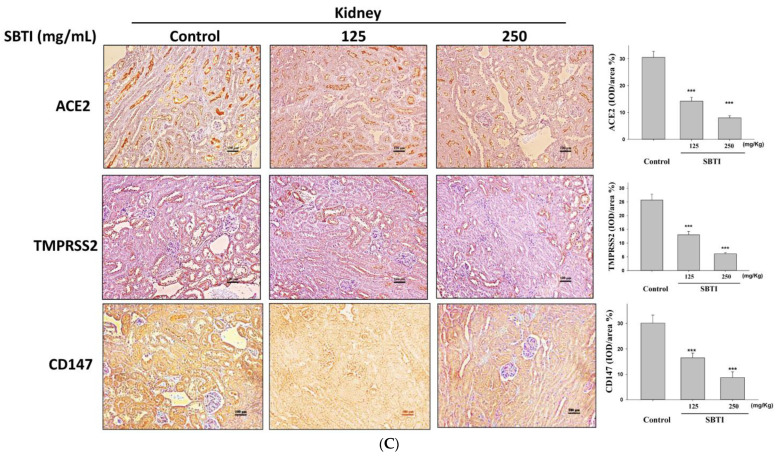
Observation of ACE2, TMPRSS2, and CD147 expression in (**A**) lungs, (**B**) livers, and (**C**) kidneys by IHC staining. The area stained in brown and red was quantified and expressed as IOD/area (%). The inhibitory effect of SBTI on ACE2, TMPRSS2, and CD147 was dose-dependent. Data are expressed as mean ± S.D. (*n* = 6). *** *p* < 0.001 compared to control. Representative microscopic images (200× magnification, scale bar 100 μm).

As shown in Figure 4, the presence of ACE2, TMPRSS2, and CD147 expression in tissues was determined by the IHC method. The control group displayed a significant number of cells stained with antibodies. A dose-dependent decrease in ACE2, TMPRSS2, and CD147 expression was observed after administration of SBTI at doses of 125 and 250 mg/kg in lung, liver, and kidney tissues. Studies suggest that SBTI can block ACE2, TMPRSS2, and CD147 expression without causing toxicity in lungs, livers, and kidneys.

### 2.5. Evaluation of the Effect of SBTI on ACE2, TMPRSS2, and CD147 Expression in Diverse Tissues

**Figure 5:**
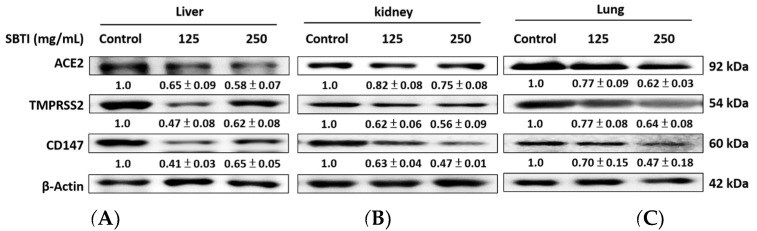
Western blot analysis was employed to quantify ACE2, TMPRSS2, and CD147 levels in mouse tissues. Close to the results of IHC staining, SBTI suppressed ACE2, TMPRSS2, and CD147 in (**A**) lungs, (**B**) livers, and (**C**) kidneys after two weeks of SBTI administration. Western blot analysis was used to quantify the protein expression of these proteins in the cells. Densitometric analysis was performed to quantify the protein bands. Each experiment was repeated independently at least three times, and representative images were shown. Each result is shown as mean ± S.D. Protein levels were quantified and displayed as fold changes relative to β-actin, which acted as the internal control for normalization.

The effect of SBTI on ACE2, TMPRSS2, and CD147 protein expression was further confirmed through Western blot analysis. By administering SBTI for 14 days, the protein expression of ACE2, TMPRSS2, and CD147 was significantly reduced in the mouse model, with dose-dependent decreases in the lungs and kidneys (Figure 5). Our findings demonstrate that SBTI is a potential drug candidate for preventing SARS-CoV-2 infection and interrupting transmission.

## 3. Discussion

Since the World Health Organization declared a global pandemic of COVID-19 on 5 May 2025, the cumulative number of confirmed cases worldwide has reached 704 million [22]. New infections and reinfections continue to be reported to date. Currently, approved therapeutic options include antivirals, immunomodulators, and monoclonal antibodies. However, monoclonal antibodies are limited by high cost and intravenous administration. Data suggest that effective and more cost-effective antiviral agents are the mainstay of preventing and controlling COVID-19, which can be administered at exposure or the onset of symptoms [23]. Moreover, COVID-19 outbreaks driven by the highly contagious Delta and Omicron variants have sparked fears of a pandemic among unvaccinated populations, as vaccine hesitancy continues despite the emergence of new deadly pathogens. Contributing factors to COVID-19 vaccine hesitancy include high distrust in government and foreign-funded health initiatives, religious beliefs, and inadequate access to medical services, despite evidence that COVID-19 vaccination is safe and effective against COVID-19 [24].

During the COVID-19 pandemic, nations have generally imposed stringent public health measures, including social distancing, mask mandates, frequent hand washing, lockdowns, and travel restrictions, to curb the spread of the virus [2]. Although these measures reduce the spread of SARS-CoV-2, they also greatly reduce people’s exposure to other common pathogens (such as the influenza virus, respiratory syncytial virus (RSV), etc.), thereby creating a phenomenon known as immune debt [25].

Specifically, the development of immune debt can be attributed to the following main reasons: 1. Decreased pathogen exposure: Social distancing and lockdown measures effectively minimize person-to-person contact, thus limiting the transmission opportunities for common respiratory and enteric pathogens. As a result, there is inadequate immune stimulation against these pathogens. Using masks and handwashing reduces the risk of pathogen transmission but also diminishes people’s natural exposure to these pathogens [26]. 2. Decline in routine vaccination rates: The COVID-19 pandemic has caused routine vaccination programs to be delayed or interrupted in many regions, leading to a decrease in herd immunity against these pathogens. Delays in vaccination result in more individuals being deprived of the immune protection they require, heightening the risk of future infections [27]. 3. Insufficient immune system stimulation. The immune system relies on repeated exposure to low-level pathogens to maintain immune memory and defense capabilities. However, anti-epidemic measures during the pandemic have reduced this exposure, leading to inadequate immune system stimulation and insufficient enhancement of immune memory [28]. 4. Decline in the level of collective immunity: With the relaxation of epidemic prevention measures and consequent re-exposure to these pathogens, the risk of infection rises. Moreover, epidemic prevention measures diminish the occurrence of natural infections, thereby reducing the level of herd immunity. With the reopening of society post-pandemic, immunocompromised groups face increased vulnerability to infection, potentially triggering disease outbreaks [28]. Public health measures during the COVID-19 pandemic inadvertently contribute to the phenomenon known as immunity debt. Although effective in mitigating COVID-19 transmission, these measures have compromised immunity against other common pathogens [29]. In light of the resumption of social interactions and the potential resurgence of pathogenic outbreaks, it is crucial to implement proactive measures aimed at addressing the challenges posed by diminished immunity. Such measures may include bolstering vaccination campaigns to enhance population-wide immunity, implementing targeted public health interventions to mitigate transmission risks, and fostering awareness regarding the importance of maintaining immunization schedules even amidst pandemic-related disruptions [30].

SARS-CoV-2 enters host cells through two primary receptors: ACE2 and CD147 [31]. The viral spike protein (SP) interacts with ACE2 or CD147 on the host cell surface, thereby facilitating viral entry and subsequent intercellular transmission. Structurally, the SP of SARS-CoV-2 is similar to that of SARS-CoV, with both exhibiting ACE2-binding capability. ACE2 has been identified as the entry point for SARS-CoV-2 into cells. It is distributed in tissues associated with multiple organ systems, encompassing the heart, blood vessels, gastrointestinal tract, lungs, kidneys, and nervous system [32]. The extensive distribution of ACE2 could account for the pathological effects and the emergence of multi-organ system diseases in patients with severe clinical outcomes. Nevertheless, organs with limited ACE2 expression still present significant tissue damage following SARS-CoV-2 infection, suggesting the potential participation of alternative receptors or accessory membrane proteins in viral entry [33]. For instance, ACE2 is expressed at relatively low levels in the lungs and is absent in blood cells, indicating potential reliance on alternative receptors for cell entry. Moreover, ACE2 expression decreases with age in humans, whereas the severity of COVID-19 tends to increase with age, further suggesting the possible involvement of alternative cellular entry pathways [34]. Moreover, during SARS-CoV-2 infection, ACE2 receptors undergo disruption, causing an imbalance in the renin–angiotensin–aldosterone system (RAAS) and angiotensin II levels. Consequently, these physiological changes elevate the risk of severe complications, particularly lung and cardiac damage, in affected patients [35]. Continued infection with SARS-CoV-2 could result in immune system imbalance and damage, which may contribute to severe complications in COVID-19 patients. Ultimately, the interaction between RAAS and ACE2 plays a crucial role in modulating host susceptibility to SARS-CoV-2 infection [36]. Additionally, the SARS-CoV-2 spike protein (SP) has been demonstrated to bind to CD147, underscoring CD147 as a viable therapeutic target for anti-SARS-CoV-2 drug development. CD147 has also been identified as a receptor for CypA [37]. Their interaction is known to contribute to cytokine storm syndromes by promoting sustained inflammation. Elevated CD147 expression activates the CD147-CypA signaling pathway, which is critically involved in inflammatory regulation via the extracellular signal-regulated kinase (ERK) pathway. Inhibition of this signaling axis has exhibited therapeutic benefits in various cancer models. Furthermore, loss of CD147 expression impairs SARS-CoV-2 entry and replication [38]. Thus, both CD147 and CypA are considered promising targets for the treatment of cancers, inflammatory disorders, and COVID-19. Currently, a Phase II clinical trial titled “Clinical Study of Anti-CD147 Humanized Meplazumab for Injection to Treat With 2019-nCoV Pneumonia” (ClinicalTrials.gov Identifier: NCT04275245) is underway in China to evaluate the efficacy of monoclonal-antibody-mediated CD147 blockade in preventing viral SP interaction and infection [39].

TMPRSS2 is a protein belonging to the serine protease family, and serine excision is known to be involved in many physiological and pathological processes. It is regulated by androgens in prostate cancer cells and also in androgen-independent prostate cancer tissue [33]. The primary route of SARS-CoV-2 entry into host cells involves the cleavage and activation of the spike protein by TMPRSS2, facilitating fusion between viral and host cell membranes. The significant contribution of TMPRSS2 to enhancing SARS-CoV-2 infectivity highlights its pivotal role in COVID-19 pathogenesis [5]. In vitro studies have revealed the inability of SARS-CoV-2 to infect ACE2-null Vero E6 and HeLa cells, emphasizing the essential role of this receptor in cellular viral entry. The membrane-bound serine protease TMPRSS2 is indispensable for cleaving ACE2 and the S protein, enabling viral entry through membrane fusion, thus playing a significant role in COVID-19 infection [29]. Camostat mesylate, a TMPRSS2 inhibitor authorized for treating pancreatitis, has been confirmed to inhibit TMPRSS2 activity and prevent the fusion of the SARS-CoV-2 virus with the host cell membrane [40]. Preventing SARS-CoV-2 entry into lung cells by inhibiting TMPRSS2, the host cell activator, and the observed therapeutic effects of camostat mesylate in critically ill COVID-19 patients indicate promising treatment directions [32].

SARS-CoV-2 antiviral drugs are classified into specific (on-target) and non-specific (off-target) types based on their physical and chemical properties. Off-target effects can cause adverse side effects and impact the drug’s efficacy and safety. Some antiviral drugs can unintentionally impact enzymes, receptors, or pathways unrelated to viral infection, leading to toxicity or unwanted immune responses. During drug development, it is essential to thoroughly investigate and minimize these off-target effects to ensure the safety and efficacy of COVID-19 treatments [41]. Research using molecular dynamics simulations found that Camostat naturally binds to the catalytic center of TMPRSS2, inhibiting its ability to proteolyze the S protein [41]. Consequently, SBTI significantly inhibited TMPRSS2 protein expression, but its mechanism is still not fully understood and needs additional research. The overall findings on ACE2 and TMPRSS2 expression levels suggest potential therapeutic approaches for COVID-19 patients.

Trypsin inhibitors are proteins belonging to a group of serine protease inhibitors found in large quantities in Leguminosae [42]. Soybeans are commonly consumed worldwide, and their seeds can contain trypsin inhibitors amounting to 6% to 8% of the total protein. Two types of trypsin inhibitors, Bowman–Birk and Kunitz, have been isolated from soybeans [43]. The SBTI utilized in this study belonged to the Kunitz type, with a molecular weight of approximately 20–22 kDa and containing two pairs of disulfide bonds [44]. These characteristics are relatively conserved in plant species, and thus Kunitz-type trypsin inhibitors have been extensively investigated for biotechnological applications with activity in mammalian pathology associated with anti-inflammatory effects, immune modulation, and possible anti-tumor activity [45,46,47]. These potential biological activities require further research to be verified. In molecular dynamics simulation research, Kunitz-type has the highest binding affinity to corona-virus envelope proteins and is the best target inhibitor protease [48]. SBTI mainly binds to trypsin to form a complex, inhibiting its digestive activity in the gastrointestinal tract. However, its inhibitory effect on trypsin will affect the digestion of nutrients in food and absorption, especially protein digestion. After hydrolysis in the gastrointestinal tract, SBTI may lose its ability to inhibit trypsin. At the same time, it may produce some new biological activities or effects, such as antioxidant capacity, anti-angiotensin activity, antigenicity, and allergenicity [49,50]. These new biological activities may differ from their original enzyme inhibitory effects.

Inhibition of ACE2 and TMPRSS2 proteins by SBTI holds promise for potentially inhibiting SARS-CoV-2 infection, but its mechanism remains unclear. Research points out that during COVID-19 infection, activated neutrophils and macrophages induce the secretion of proteases (neutrophil elastase and matrix metalloproteinases) to help the virus enter or regulate infected cells. Plant protease inhibitors added in vitro can easily target cell surface proteases essential for viral attachment and infection, though they may not reach the intracellular compartments at levels high enough to interfere with the viral life cycle [51]. Protease inhibitors can interfere with the ability of viruses to enter host cells by inhibiting key proteases required for viral entry and replication. There are studies in the literature screening seven different sources of plant protease inhibitors: cystatin-I, eravatmin, pumpkin, Kunitz, Bowman–Birk, α-amylase inhibitor, and potato serine protease inhibitor, among which Kunitz, α-amylase, and pumpkin protease inhibitors show the largest binding energy. To visualize the motion and stability of the docked complex with the lowest binding energy, molecular dynamics simulations followed by normal mode analysis were performed. Plant protease inhibitors present promising targets against emerging coronavirus strains due to their natural origin and fewer side effects compared to synthetic compounds [51,52]. However, specific evidence linking hydrolyzed SBTI directly to anti-COVID-19 activity remains limited. Therefore, further studies using protease inhibitors to inhibit the SARS-CoV-2 replication cycle are needed.

SBTI’s trypsin-inhibitory activity may potentially influence nutrient digestion. Among the various processing strategies, heat treatment is widely regarded as the most effective approach to enhance the nutritional value of pulse seeds. This is primarily due to its ability to improve protein digestibility by inactivating heat-labile anti-nutritional factors, especially protease inhibitors. Heat treatment induces cleavage of intermolecular bonds that maintain the tertiary structure of these inhibitors, leading to conformational changes at their active sites. Moreover, heat treatment is the most commonly employed method for processing pulse grains, both in traditional households and at industrial scales, owing to its effectiveness in improving palatability. Common heat-processing techniques include boiling, steaming, autoclaving, microwaving, and roasting. SBTI is known to be heat-sensitive and is completely inactivated at approximately 108 °C after 15 to 30 min of exposure. In soybeans, various heat treatments—such as boiling at 100 °C for 9 min, roasting for 2 min, steaming for 7 to 30 min, microwaving for 3 min, and autoclaving at 121 °C for 15 min—effectively inactivate most trypsin inhibitor activities (TIAs) [53]. In the present study, oral administration of SBTI for 14 consecutive days did not cause significant changes in body weight compared to the untreated group. Histopathological examination further confirmed the absence of toxicity in the lungs, liver, and kidneys. Notably, standard antiviral regimens for COVID-19 typically last ≤14 days, which is substantially shorter than the duration required for the onset of nutritional toxicity. In animal models, pancreatic hyperplasia has only been observed following continuous intake of trypsin inhibitors for periods exceeding 8 weeks. Taken together, the dual safety features of SBTI—namely, its thermal lability and favorable short-term safety profile—highlight its potential as a promising antiviral candidate. Its derivation from agricultural byproducts and suitability for safe use in short-term antiviral therapy positions SBTI as a compelling biomedical solution with resource-circulating value, particularly relevant for implementation in resource-limited settings.

Cytokine storms significantly contribute to the pathogenesis of COVID-19 and may be linked to disease severity and mortality. Ulinastatin, a urinary trypsin inhibitor, has shown efficacy in inhibiting a variety of cellular proteolytic enzymes and has a broad therapeutic mechanism [54]. Ulinastatin has been proven to inhibit the production of inflammatory cytokines and adhesion molecules. In addition, it can enhance the stability of the lysosomal membrane and reduce the synthesis and transport of lysosomal enzymes, thus scavenging oxygen or hydroxyl radicals [55]. Studies have confirmed that supplementing the diet with SBTI can effectively reduce inflammation by inhibiting the activation of the MAP kinase pathway, including ERK1/2, JNK, and p38, and subsequently decreasing the expression of cytokines TNF-α, IL-1β, and IL-6 in mice [56]. Dietary supplementation with SBTI may be highly beneficial for patients with inflammation because SBTI is readily available and inexpensive from soybeans. Notably, the MAPK/ERK and NF-κB signaling pathways exhibit cross-regulatory interactions that co-modulate the transcription of ACE2, TMPRSS2, and CD147—key molecules involved in viral entry and inflammation, including in SARS-CoV-2 infection [57]. Upon stimulation by proinflammatory cytokines such as TNF-α and IL-1β, the NF-κB pathway directly binds to the promoter regions of ACE2 and CD147, significantly upregulating their expression in pulmonary and intestinal epithelial cells. Concurrently, it indirectly enhances TMPRSS2 transcription via androgen receptor (AR) activation [58]. In parallel, the MAPK/ERK cascade phosphorylates transcription factors AP-1 and SP1 to promote ACE2 expression and activates AR to facilitate TMPRSS2 transcription. This pathway also induces the secretion of matrix metalloproteinases (MMPs), establishing a CD147-driven positive feedback loop that accelerates viral invasion [59]. Substantial crosstalk exists between these pathways. ERK-mediated phosphorylation of IKK activates NF-κB, thereby amplifying the expression of ACE2, TMPRSS2, and CD147. Moreover, SARS-CoV-2 activates the TLR–NF-κB/MAPK axis, synergistically upregulating ACE2, TMPRSS2, and CD147, resulting in a “three-target enhancement” effect. This not only facilitates viral replication but also contributes to the cytokine storm observed in severe cases. Therefore, targeting the MAPK/ERK–NF-κB regulatory network offers a promising strategy for antiviral and anti-inflammatory therapies. Pharmacological inhibition of NF-κB or ERK effectively reduces ACE2, TMPRSS2, and CD147 expression, thereby impeding viral entry. Collectively, these findings underscore the therapeutic potential of modulating MAPK/ERK and NF-κB signaling to combat viral infection and its associated pathologies.

Based on the preceding discussion, we utilized SBTI as the research material and carried out in vitro and in vivo experiments to examine the effects on ACE2, TMPRSS2, and CD147. To focus on the effects on organs other than the lungs, two cellular models commonly used in SARS-CoV-2 studies were used in this study, namely, the HepG2 cell line and the HEK293T cell line [60]. According to our results, the administration of SBTI at doses of 125 and 250 μg/mL resulted in cell viability greater than 80% and achieved a decrease in the performance of ACE2, TMPRSS2, and CD147. Studies using mouse models have made vaccines and various therapeutic regimens against SARS-CoV-2 possible [61,62]. In this study, mice were given SBTI for two weeks, and the expression of ACE2, TMPRSS2, and CD147 in lungs, livers, and kidneys was observed by IHC staining and the Western blot method. The H&E results demonstrated that SBTI did not cause damage to the lungs, liver, or kidneys, while IHC staining data showed that SBTI dose-dependently inhibited the expression of ACE2, TMPRSS2, and CD147 in tissues. In conclusion, SBTI has shown potential to inhibit key proteins in vivo and in vitro studies, which may be a potential ingredient for further drug or functional food studies to inhibit virus replication and transmission.

## 4. Materials and Methods

### 4.1. Cell Culture and Viability Assay

Human HepG2 (hepatocellular carcinoma cell line) was provided by the Biological Resources Collection and Research Center (BCRC; Hsinchu, Taiwan) (BCRC number: 60364). The HEK293T (human embryonic kidney normal cell line) cell line was obtained from Hsien-Tsung Yao (Department of Nutrition, China Medical University, Taichung, Taiwan). Human Calu-3 cells, derived from bronchial adenocarcinoma submucosal glands, were obtained from Ubigene Biosciences Co., Ltd. (Guangzhou, China). HepG2 and HEK293T cell lines were grown in Dulbecco’s modified Eagle’s medium supplemented with 10% FBS and cultured at 37 °C and 5% CO_2_. When the cells reached a specific density, the seeding density for HepG2 and HEK293T cells in 96-well plates was 2.5 × 10^4^ cells per well, respectively. Calu-3 cells were cultured in MEM medium supplemented with 10% fetal bovine serum, 100 IU/mL penicillin, and 100 μg/mL streptomycin under standard conditions (37 °C, 5% CO_2_). After the cells were attached, a trypsin inhibitor from *Glycine max* (SBTI) (T-9128; Sigma-Aldrich, Saint Louis, MI, USA) dissolved in PBS was added to the culture medium for 24 h. A total of 100 μL of MTT reagent (HY-15924; MedChem Express, Monmouth Junction, NJ, USA) was added to each well, and the mixture was incubated for 3 h. The absorbance at 570 nm was measured using an ELISA reader to determine cell viability (Molecular Devices, Taipei, Taiwan).

### 4.2. Western Blot Analysis

In 6-well plates, HepG2, HEK293T, and Calu-3 cells were plated at a density of 2.5 × 10^4^ cells per well. After the cells were attached, SBTI dissolved in PBS was supplemented into the culture medium, and the proteins were collected by RIPA buffer after 24 h of treatment. The supernatant was then subjected to centrifugation at 15,000 rpm and 4 °C for 15 min. For mouse tissues, an appropriate size was cut, and 10-fold of the RIPA buffer was added; following this, the mixture was homogenized with a homogenizer. The supernatant was collected by centrifugation for 15 min at 15,000 rpm and four °C. The supernatant was stored at −20 °C for subsequent processing. Measurement of the total protein concentration was conducted employing the Bio-Rad Protein Assay Kit (Bio-Rad, Hercules, CA, USA). A total of 10 μg of protein was separated by 12% SDS polyacrylamide gel electrophoresis and transferred to a membrane. Primary antibodies ACE2 (GTX101395; Genetex, San Antonio, TX, USA), TMPRSS2 (GTX100743; Genetex, San Antonio, TX, USA), and CD147 (R381696; Zen-BioScience, Chengdu, China) were incubated at 4 °C overnight. Secondary antibodies (anti-rabbit IgG antibody: ARG65351; Arigo, Hsinchu, Taiwan) and horseradish peroxidase conjugate with ECL substrate (201765; Merck, NJ, USA) were added on alternate days. The results were photographed using the MultiGel-21 system (TOPBIO, New Taipei city, Taiwan), and the optical densities of the strips were analyzed with AlphaEaseFC 4.0 software (Alpha Innotech Corporation, San Leandro, CA, USA).

### 4.3. Mouse Experiment

Female C57BL/6 mice (BioLASCO, Taipei, Taiwan), with weights ranging from 18 to 20 g and aged 6–8 weeks, were randomly assigned to a control group and 2 dose groups (low and high dose) (*n* = 6). The control group was raised as usual under standard animal husbandry practices. In the administration group, SBTI dissolved in reverse osmosis water was fed by oral gavage at doses of 125 mg/kg or 250 mg/kg for 14 consecutive days. The mice were weighed on days 0, 7, and 14. The mice were fasted on the night of the 14th and executed the next day; the tissues were collected for further analysis.

### 4.4. Hematoxylin and Eosin (H&E) Staining

After the sacrifice, the organs of the lungs, liver, and kidneys were sectioned and embedded in paraffin wax. After being dewaxed and dehydrated, staining with hematoxylin was conducted to visualize the nuclei in the sections and then stained with eosin for secondary staining. After being dehydrated and cleared, the H&E staining results were visualized through microscopy (ECLIPSE TS100; Nikon, Tokyo, Japan) and photographed and recorded with a microscopic camera (ProgRes camera; Jenoptik AG, Jena, Germany).

### 4.5. Immunohistochemistry (IHC) Staining

Sections of lungs, livers, and kidneys removed after sacrifice were incubated with ACE2 primary antibody (bs-1004R diluted 50×; Bioss, Woburn, MA, USA), TMPRSS2 primary antibody (ab214462 diluted 200×; Abcam, Cambridge, UK), or CD147 primary antibody (R381696 diluted 50×; Zen-BioScience, Chengdu, China), respectively. IHC evaluation was performed using the Polink-2 Plus HRP Rabbit DAB Detection Kit (D39; OriGene, Rockville, MD, USA) according to the recommended protocol from the manufacturer, and microscopic examination of the results was conducted using a microscope (ECLIPSE TS100; Nikon, Japan), with images captured with a microscope camera (ProgRes camera; Jenoptik AG, Germany).

### 4.6. Statistical Analysis

All data were at least tripled and shown as mean ± standard error (S.D.). Analysis was performed using SPSS software version 22.0. (SPSS Inc., Chicago, IL, USA). Analyses were performed by one-way analysis of variance, and post hoc tests were confirmed by Scheffé’s. A *p*-value less than 0.05 was considered statistically significant.

## 5. Conclusions

The global outbreak of SARS-CoV-2 has significantly increased consumer awareness regarding the importance of maintaining health through diet, thereby intensifying interest in functional foods and bioactive ingredients capable of supporting physiological regulation and enhancing immune defense. SBTI, known for its diverse biological activities—including anti-carcinogenic and anti-inflammatory effects—has attracted considerable attention as a promising candidate for application in both functional food products and pharmaceutical formulations. This study investigated the antiviral potential of SBTI through comprehensive in vitro and in vivo analyses. The results demonstrated that SBTI effectively combats viral invasion by downregulating the expression of key host entry proteins, namely, ACE2, CD147, and TMPRSS2, which are critical for SARS-CoV-2 entry into host cells. Given these findings, SBTI emerges as a potential natural antiviral agent with clinical relevance in the management of COVID-19. However, to substantiate its therapeutic efficacy and safety, rigorously designed clinical trials are urgently warranted. Future investigations should focus on pharmacokinetics, dosage optimization, long-term safety, and potential synergistic effects with existing treatments, thereby paving the way for its translational application in clinical practice.

## Data Availability

Data are contained within the article.

## References

[1] Halawa S., Pullamsetti S.S., Bangham C.R.M., Stenmark K.R., Dorfmuller P., Frid M.G., Butrous G., Morrell N.W., de Jesus Perez V.A., Stuart D.I. (2022). Potential long-term effects of SARS-CoV-2 infection on the pulmonary vasculature: A global perspective. Nat. Rev. Cardiol..

[2] Tregoning J.S., Flight K.E., Higham S.L., Wang Z., Pierce B.F. (2021). Progress of the COVID-19 vaccine effort: Viruses, vaccines and variants versus efficacy, effectiveness and escape. Nat. Rev. Immunol..

[3] Wong S.C., Chan V.W., Yuen L.L., AuYeung C.H., Leung J.O., Li C.K., Kwok M.O., So S.Y., Chen J.H., Chiu K.H. (2023). Infection of healthcare workers despite a high vaccination rate during the fifth wave of COVID-19 due to Omicron variant in Hong Kong. Infect. Prev. Pract..

[4] Choudhary S., Sreenivasulu K., Mitra P., Misra S., Sharma P. (2021). Role of Genetic Variants and Gene Expression in the Susceptibility and Severity of COVID-19. Ann. Lab. Med..

[5] Lanjanian H., Moazzam-Jazi M., Hedayati M., Akbarzadeh M., Guity K., Sedaghati-Khayat B., Azizi F., Daneshpour M.S. (2021). SARS-CoV-2 infection susceptibility influenced by ACE2 genetic polymorphisms: Insights from Tehran Cardio-Metabolic Genetic Study. Sci. Rep..

[6] Su Y.C., Huang G.J., Lin J.G. (2022). Chinese herbal prescriptions for COVID-19 management: Special reference to Taiwan Chingguan Yihau (NRICM101). Front. Pharmacol..

[7] Devaux C.A., Rolain J.M., Raoult D. (2020). ACE2 receptor polymorphism: Susceptibility to SARS-CoV-2, hypertension, multi-organ failure, and COVID-19 disease outcome. J. Microbiol. Immunol. Infect..

[8] Abbasi A.Z., Kiyani D.A., Hamid S.M., Saalim M., Fahim A., Jalal N. (2021). Spiking dependence of SARS-CoV-2 pathogenicity on TMPRSS2. J. Med. Virol..

[9] Huang J., Song W., Huang H., Sun Q. (2020). Pharmacological Therapeutics Targeting RNA-Dependent RNA Polymerase, Proteinase and Spike Protein: From Mechanistic Studies to Clinical Trials for COVID-19. J. Clin. Med..

[10] Luan B., Huynh T., Cheng X., Lan G., Wang H.R. (2020). Targeting Proteases for Treating COVID-19. J. Proteome Res..

[11] Ou X., Liu Y., Lei X., Li P., Mi D., Ren L., Guo L., Guo R., Chen T., Hu J. (2021). Author Correction: Characterization of spike glycoprotein of SARS-CoV-2 on virus entry and its immune cross-reactivity with SARS-CoV. Nat. Commun..

[12] Bojkova D., McGreig J.E., McLaughlin K.-M., Masterson S.G., Widera M., Krähling V., Ciesek S., Wass M.N., Michaelis M., Jindrich Cinatl J. (2020). SARS-CoV-2 and SARS-CoV differ in their cell tropism and drug sensitivity profiles. bioRxiv.

[13] Shouman S., El-Kholy N., Hussien A.E., El-Derby A.M., Magdy S., Abou-Shanab A.M., Elmehrath A.O., Abdelwaly A., Helal M., El-Badri N. (2024). SARS-CoV-2-associated lymphopenia: Possible mechanisms and the role of CD147. Cell Commun. Signal..

[14] Cavezzi A., Menicagli R., Troiani E., Corrao S. (2022). COVID-19, Cation dysmetabolism, sialic acid, CD147, ACE2, viroporins, hepcidin and ferroptosis: A possible unifying hypothesis. F1000Research.

[15] Fang E.F., Wong J.H., Ng T.B. (2010). Thermostable Kunitz trypsin inhibitor with cytokine inducing, antitumor and HIV-1 reverse transcriptase inhibitory activities from Korean large black soybeans. J. Biosci. Bioeng..

[16] Fang E.F., Wong J.H., Bah C.S., Lin P., Tsao S.W., Ng T.B. (2010). Bauhinia variegata var. variegata trypsin inhibitor: From isolation to potential medicinal applications. Biochem. Biophys. Res. Commun..

[17] Luz A.B.S., de Medeiros A.F., Bezerra L.L., Lima M.S.R., Pereira A.S., EGO E.S., Passos T.S., Monteiro N.K.V., Morais A.H.A. (2023). Prospecting native and analogous peptides with anti-SARS-CoV-2 potential derived from the trypsin inhibitor purified from tamarind seeds. Arab. J. Chem..

[18] Gupta S., Kanwar S.S. (2021). Plant protease inhibitors and their antiviral activities-Potent therapeutics for SARS-CoV-2. J. Med. Discov..

[19] Kobayashi H. (2013). Prevention of cancer and inflammation by soybean protease inhibitors. Front Biosci.

[20] Pires De Souza G.A., Le Bideau M., Boschi C., Wurtz N., Colson P., Aherfi S., Devaux C., La Scola B. (2022). Choosing a cellular model to study SARS-CoV-2. Front. Cell Infect. Microbiol..

[21] Smirnova O.A., Ivanova O.N., Fedyakina I.T., Yusubalieva G.M., Baklaushev V.P., Yanvarev D.V., Kechko O.I., Mitkevich V.A., Vorobyev P.O., Fedorov V.S. (2023). SARS-CoV-2 Establishes a Productive Infection in Hepatoma and Glioblastoma Multiforme Cell Lines. Cancers.

[22] WHO COVID-19 Dashboard. https://data.who.int/dashboards/covid19/cases?n=c.

[23] Mitja O., Clotet B. (2020). Use of antiviral drugs to reduce COVID-19 transmission. Lancet Glob. Health.

[24] Lin J.G., Huang G.J., Su Y.C. (2023). Efficacy analysis and research progress of complementary and alternative medicines in the adjuvant treatment of COVID-19. J. Biomed. Sci..

[25] Vasquez-Bonilla W.O., Orozco R., Argueta V., Sierra M., Zambrano L.I., Muñoz-Lara F., López-Molina D.S., Arteaga-Livias K., Grimes Z., Bryce C. (2020). A review of the main histopathological findings in coronavirus disease 2019. Hum. Pathol..

[26] Munro A.P., Jones C.E. (2022). Immunity debt and unseasonal childhood respiratory viruses. Br. J. Hosp. Med..

[27] Billard M.N., Bont L.J. (2023). Quantifying the RSV immunity debt following COVID-19: A public health matter. Lancet Infect. Dis..

[28] Principi N., Autore G., Ramundo G., Esposito S. (2023). Epidemiology of Respiratory Infections during the COVID-19 Pandemic. Viruses.

[29] Yang M.C., Su Y.T., Chen P.H., Tsai C.C., Lin T.I., Wu J.R. (2023). Changing patterns of infectious diseases in children during the COVID-19 pandemic. Front. Cell Infect. Microbiol..

[30] Rubin R. (2024). From “Immunity Debt” to “Immunity Theft”-How COVID-19 Might Be Tied to Recent Respiratory Disease Surges. JAMA.

[31] Bourgonje A.R., Abdulle A.E., Timens W., Hillebrands J.L., Navis G.J., Gordijn S.J., Bolling M.C., Dijkstra G., Voors A.A., Osterhaus A.D. (2020). Angiotensin-converting enzyme 2 (ACE2), SARS-CoV-2 and the pathophysiology of coronavirus disease 2019 (COVID-19). J. Pathol..

[32] Li Y., Zhou W., Yang L., You R. (2020). Physiological and pathological regulation of ACE2, the SARS-CoV-2 receptor. Pharmacol. Res..

[33] Raghav P.K., Kalyanaraman K., Kumar D. (2021). Human cell receptors: Potential drug targets to combat COVID-19. Amino Acids.

[34] Chien L.H., Deng J.S., Jiang W.P., Chen C.C., Chou Y.N., Lin J.G., Huang G.J. (2022). Study on the potential of Sanghuangporus sanghuang and its components as COVID-19 spike protein receptor binding domain inhibitors. Biomed. Pharmacother..

[35] Zhang Q., Xiang R., Huo S., Zhou Y., Jiang S., Wang Q., Yu F. (2021). Molecular mechanism of interaction between SARS-CoV-2 and host cells and interventional therapy. Signal Transduct. Tar..

[36] Hoffmann M., Hofmann-Winkler H., Smith J.C., Kruger N., Arora P., Sorensen L.K., Sogaard O.S., Hasselstrom J.B., Winkler M., Hempel T. (2021). Camostat mesylate inhibits SARS-CoV-2 activation by TMPRSS2-related proteases and its metabolite GBPA exerts antiviral activity. EBioMedicine.

[37] Ren H.L., Wen G.M., Zhao Z.Y., Liu D.H., Xia P. (2022). Can CD147 work as a therapeutic target for tumors through COVID-19 infection?. Int. J. Med. Sci..

[38] Behl T., Kaur I., Aleya L., Sehgal A., Singh S., Sharma N., Bhatia S., Al-Harrasi A., Bungau S. (2022). CD147-spike protein interaction in COVID-19: Get the ball rolling with a novel receptor and therapeutic target. Sci. Total Environ..

[39] Wu J., Chen L., Qin C., Huo F., Liang X., Yang X., Zhang K., Lin P., Liu J., Feng Z. (2022). CD147 contributes to SARS-CoV-2-induced pulmonary fibrosis. Signal Transduct. Target. Ther..

[40] Sakr Y., Bensasi H., Taha A., Bauer M., Ismail K., Belhaj G., Afet K.M., Munde D., Monk D., UAE-Jena Research Group (2021). Camostat mesylate therapy in critically ill patients with COVID-19 pneumonia. Intensive Care Med..

[41] Zhu H., Du W., Song M., Liu Q., Herrmann A., Huang Q. (2020). Spontaneous binding of potential COVID-19 drugs (Camostat and Nafamostat) to human serine protease TMPRSS2. Comput. Struct. Biotechnol. J..

[42] Oliva M.L., Ferreira Rda S., Ferreira J.G., de Paula C.A., Salas C.E., Sampaio M.U. (2011). Structural and functional properties of kunitz proteinase inhibitors from leguminosae: A mini review. Curr. Protein Pept. Sci..

[43] Roosta H.R., Javadi T., Nazari F. (2011). Isolation and characterization of trypsin inhibitors (Kunitz soybean trypsin inhibitor, Bowman-Birk inhibitor) in soybean. Adv. Environ. Biol..

[44] Bendre A.D., Ramasamy S., Suresh C.G. (2018). Analysis of Kunitz inhibitors from plants for comprehensive structural and functional insights. Int. J. Biol. Macromol..

[45] Bonturi C.R., Silva Teixeira A.B., Rocha V.M., Valente P.F., Oliveira J.R., Filho C.M.B., Fátima Correia Batista I., Oliva M.L.V. (2022). Plant Kunitz inhibitors and their interaction with proteases: Current and potential pharmacological targets. Int. J. Mol. Sci..

[46] Flavin D.F. (1982). The effects of soybean trypsin inhibitors on the pancreas of animals and man: A review. Vet. Hum. Toxicol..

[47] Cid-Gallegos M.S., Corzo-Ríos L.J., Jiménez-Martínez C., Sánchez-Chino X.M. (2022). Protease inhibitors from plants as therapeutic agents- a review. Plant Foods Hum. Nutr..

[48] Kennedy A.R. (2023). Proteases, protease inhibitors and radiation carcinogenesis. Int. J. Radiat. Biol..

[49] Nath A., Ahmad A.S., Amankwaa A., Csehi B., Mednyánszky Z., Szerdahelyi E., Tóth A., Tormási J., Truong D.H., Abrankó L. (2022). Hydrolysis of soybean milk protein by papain: Antioxidant, anti-Angiotensin, antigenic and digestibility perspectives. Bioengineering.

[50] De La Barca A.M., Wall A., López-Díaz J.A. (2005). Allergenicity, trypsin inhibitor activity and nutritive quality of enzymatically modified soy proteins. Int. J. Food Sci. Nutr..

[51] Visser N., Herreman L.C.M., Vandooren J., Pereira R.V.S., Opdenakker G., Spelbrink R.E.J., Wilbrink M.H., Bremer E., Gosens R., Nawijn M.C. (2024). Novel high-yield potato protease inhibitor panels block a wide array of proteases involved in viral infection and crucial tissue damage. J. Mol. Med..

[52] Kirar M., Singh H., Sehrawat N. (2022). Virtual screening and molecular dynamics simulation study of plant protease inhibitors against SARS-CoV-2 envelope protein. Informatics Med. Unlocked.

[53] Avilés-Gaxiola S., Chuck-Hernández C., Saldívar S.O.S. (2018). Inactivation methods of trypsin inhibitor in legumes: A review. J. Food Sci..

[54] Jain A., Kasliwal R., Jain S.S., Jain R., Gupta D., Gupta P., Jain A., Tambi R., Panwar P., Meena M. (2022). Effect of urinary trypsin inhibitor (ulinastatin) therapy in COVID-19. Indian J. Crit. Care Med..

[55] Mehta Y., Zirpe K., Dixit S., Ansari A., Mehta C., Deshmukh A., Ambapkar S., Ambapkar S., Joshi M., Joshi A. (2023). Ulinastatin add-on to standard of care in critically Ill COVID-19 patients: A multicenter, retrospective study. J. Assoc. Physicians India..

[56] Wu C.Y., Lin Y.S., Yang Y.H., Shu L.H., Cheng Y.C., Liu H.T. (2020). GB-2 inhibits ACE2 and TMPRSS2 expression: In vivo and in vitro studies. Biomed. Pharmacother..

[57] Rasmi Y., Hatamkhani S., Naderi R., Shokati A., Zadeh V.N., Hosseinzadeh F., Farnamian Y., Jalali L. (2022). Molecular signaling pathways, pathophysiological features in various organs, and treatment strategies in SARS-CoV2 infection. Acta Histochem..

[58] Qiao Y., Wang X.-M., Mannan R., Pitchiaya S., Zhang Y., Wotring J.W., Xiao L., Robinson D.R., Wu Y.-M., Tien J.C.-Y. (2021). Targeting transcriptional regulation of SARS-CoV-2 entry factors ACE2 and TMPRSS2. Proc. Natl. Acad. Sci. USA.

[59] Schwartz J., Capistrano K.J., Gluck J., Hezarkhani A., Naqvi A.R. (2024). SARS-CoV-2, periodontal pathogens, and host factors: The trinity of oral post-acute sequelae of COVID-19. Rev. Med. Virol..

[60] Jiang W.P., Deng J.S., Yu C.C., Lin J.G., Huang G.J. (2024). Anti-SARS-CoV-2 viral activity of sweet potato trypsin inhibitor via downregulation of TMPRSS2 Activity and ACE2 expression in vitro and in vivo. Int. J. Mol. Sci..

[61] Sun T.K., Huang W.C., Sun Y.W., Deng J.S., Chien L.H., Chou Y.N., Jiang W.P., Lin J.G., Huang G.J. (2022). *Schizophyllum commune* Reduces Expression of the SARS-CoV-2 Receptors ACE2 and TMPRSS2. Int. J. Mol. Sci..

[62] Chen Y.R., Jiang W.P., Deng J.S., Chou Y.N., Wu Y.B., Liang H.J., Lin J.G., Huang G.J. (2023). *Anisomeles indica* extracts and their constituents suppress the protein expression of ACE2 and TMPRSS2 in vivo and in vitro. Int. J. Mol. Sci..

